# Supplementary material for: Multi‐dataset identification of innovative feature genes and molecular mechanisms in keratoconus
Source: J Cell Mol Med. 2024 Sep 19;28(18):e70079. doi: 10.1111/jcmm.70079 (PMC11412914; doi:10.1111/jcmm.70079)
Supplement: Supplementary file 2 — Material S2. [file JCMM-28-e70079-s002.zip › 7.GSEA analysis/2.ATOH7/KEGG_BETA_ALANINE_METABOLISM.html]

Details for gene set KEGG\_BETA\_ALANINE\_METABOLISM[GSEA]

|  || Dataset | ATOH7.group.cls#h\_versus\_l |
| Phenotype | group.cls#h\_versus\_l |
| Upregulated in class | l |
| GeneSet | KEGG\_BETA\_ALANINE\_METABOLISM |
| Enrichment Score (ES) | -0.25483376 |
| Normalized Enrichment Score (NES) | -0.7261637 |
| Nominal p-value | 0.8608696 |
| FDR q-value | 1.0 |
| FWER p-Value | 1.0 |
Table: GSEA Results Summary

  

Fig 1: Enrichment plot: KEGG\_BETA\_ALANINE\_METABOLISM      
 Profile of the Running ES Score & Positions of GeneSet Members on the Rank Ordered List

  

| SYMBOL | TITLE | RANK IN GENE LIST | RANK METRIC SCORE | RUNNING ES | CORE ENRICHMENT || 1 | ALDH2 | na | 221 | 0.659 | 0.1912 | Yes |
| 2 | AOC3 | na | 3743 | 0.247 | 0.1636 | Yes |
| 3 | GAD1 | na | 4303 | 0.225 | 0.2149 | Yes |
| 4 | DPYD | na | 5776 | 0.177 | 0.2256 | Yes |
| 5 | ALDH9A1 | na | 7741 | 0.125 | 0.2063 | Yes |
| 6 | ALDH1B1 | na | 7850 | 0.122 | 0.2397 | Yes |
| 7 | EHHADH | na | 8475 | 0.109 | 0.2543 | Yes |
| 8 | SRM | na | 11469 | 0.059 | 0.1858 | No |
| 9 | MLYCD | na | 13163 | 0.039 | 0.1486 | No |
| 10 | SMS | na | 13284 | 0.037 | 0.1564 | No |
| 11 | ALDH7A1 | na | 14436 | 0.026 | 0.1311 | No |
| 12 | DPYS | na | 16196 | 0.011 | 0.0838 | No |
| 13 | HADHA | na | 16925 | 0.005 | 0.0644 | No |
| 14 | ECHS1 | na | 16985 | 0.005 | 0.0641 | No |
| 15 | ACADM | na | 20219 | -0.024 | -0.0219 | No |
| 16 | ALDH3A2 | na | 20819 | -0.030 | -0.0302 | No |
| 17 | ABAT | na | 23756 | -0.066 | -0.0950 | No |
| 18 | HIBCH | na | 24320 | -0.074 | -0.0890 | No |
| 19 | UPB1 | na | 25229 | -0.089 | -0.0884 | No |
| 20 | GAD2 | na | 31008 | -0.247 | -0.1807 | No |
| 21 | CNDP1 | na | 32766 | -0.348 | -0.1271 | No |
| 22 | AOC2 | na | 34386 | -0.613 | 0.0099 | No |
Table: GSEA details [plain text format]

  

Fig 2: KEGG\_BETA\_ALANINE\_METABOLISM      
 Blue-Pink O' Gram in the Space of the Analyzed GeneSet

  

Fig 3: KEGG\_BETA\_ALANINE\_METABOLISM: Random ES distribution      
 Gene set null distribution of ES for **KEGG\_BETA\_ALANINE\_METABOLISM**
